# Effects of the low carbohydrate, high fat diet on glycemic control and body weight in patients with type 2 diabetes: experience from a community-based cohort

**DOI:** 10.1136/bmjdrc-2019-000980

**Published:** 2020-03-18

**Authors:** Shabina Roohi Ahmed, Sridevi Bellamkonda, Mihail Zilbermint, Jiangxia Wang, Rita Rastogi Kalyani

**Affiliations:** 1 Department of Medicine, Division of Endocrinology, Diabetes and Metabolism, Johns Hopkins University School of Medicine, Baltimore, Maryland, USA; 2 Endocrinology, Johns Hopkins Community Physicians, Bethesda, Maryland, USA; 3 Department of Medicine, Division of General Internal Medicine, Johns Hopkins University School of Medicine, Baltimore, Maryland, USA; 4 Internal Medicine, Johns Hopkins Community Physicians, Germantown, Maryland, USA; 5 Endocrinology, Diabetes and Metabolism, Johns Hopkins Community Physicians Suburban Hospital, Suburban Hospital, Bethesda, Maryland, USA; 6 Biostatistics Consulting Center, Johns Hopkins University Bloomberg School of Public Health, Baltimore, Maryland, USA

**Keywords:** type 2 diabetes, nutritional management, dietary intervention

## Abstract

**Objective:**

The optimal diet to improve glycemia in patients with type 2 diabetes remains unclear. Low carbohydrate, high fat (LCHF) diets can improve glycemic control, but have not been investigated in real-world settings.

**Research design and methods:**

We investigated effects of the LCHF diet compared with usual care in a community-based cohort of patients with type 2 diabetes by performing a retrospective study of 49 patients who followed the LCHF diet for ≥3 months, and compared glycemic outcomes with age-matched and body mass index (BMI)-matched controls who received usual care (n=75). The primary outcome was change in A1C from baseline to the end of follow-up.

**Results:**

Compared with the usual care group, the LCHF group showed a significantly greater reduction in A1C (−1.29% (95% CI −1.75 to −0.82; p<0.001)) and body weight (−12.8 kg (95% CI −14.7 to −10.8; p<0.001) at the end of follow-up after adjusting for age, sex, baseline A1C, BMI, baseline insulin dose. Of the patients initially taking insulin therapy in the LCHF group, 100% discontinued it or had a reduction in dose, compared with 23.1% in the usual care group (p<0.001). The LCHF group also had significantly greater reduction in fasting plasma glucose (−43.5 vs −8.5 mg/mL; p=0.03) compared with usual care.

**Conclusions:**

In a community-based cohort of type 2 diabetes, the LCHF diet was associated with superior A1C reduction, greater weight loss and significantly more patients discontinuing or reducing antihyperglycemic therapies suggesting that the LCHF diet may be a metabolically favorable option in the dietary management of type 2 diabetes.

**Significance of this study**

**What is already known about this subject?**

- Prior studies indicate that low carbohydrate, high fat (LCHF) diets have the potential to improve glycemic control and result in reduction of glucose lowering medications.

**What are the new findings?**

- To our knowledge, this is the first study to date that investigates the implementation of the low carbohydrate, high fat (LCHF) diet in a community-based setting, making the results generalizable and applicable to the clinical practitioner.
- Our study shows that it is feasible and safe to implement the LCHF diet in a ‘real-world’ community practice setting among patients with type 2 diabetes, and that this diet may offer superior glycemic reduction, along with greater weight loss, compared with usual care over 3 months.
- All patients following the LCHF diet who initially took insulin had either a reduction or discontinuation of this therapy by their healthcare provider when clinically indicated, compared with less than a quarter of those receiving usual care.

**How might these results change the focus of research or clinical practice?**

- For motivated patients, the LCHF diet should be considered as a viable treatment option for type 2 diabetes.
- Future research questions include:
  - What patient characteristics are predictive of greater levels of glycemic reduction utilizing the LCHF diet?
  - What are the long-term effects of the LCHF diet on cardiovascular risk factors in patients with type 2 diabetes?
  - What is the optimal proportion of carbohydrates in the LCHF diet for weight loss and cardiovascular benefit?
  - Is the LCHF diet sustainable in real-world settings over long periods of time (ie, >1 year)?

## INTRODUCTION

Approximately two-thirds of the US population is overweight or obese,^1 2^ which is also linked to the rising numbers of people with type 2 diabetes. In 2017, approximately 30.3 million people in the USA were living with type 2 diabetes,^3^ and projections suggest up to one-third of Americans will be diagnosed with diabetes by 2050.^4^ Modest weight loss with lifestyle changes may prevent progression of prediabetes to type 2 diabetes,^5^ and improve glycemic control in patients with type 2 diabetes.^6^

The standard for treatment of type 2 diabetes is dietary modification, regular physical activity and, for most patients, the use of antihyperglycemic medications. The rising prevalence of type 2 diabetes suggests that conventional therapy for type 2 diabetes may be inadequate. Part of the difficulty is that recommending and successfully implementing dietary changes, in practice, can be quite challenging for patients and healthcare providers alike. The optimal diet to achieve weight loss and improve glycemic control remains unclear, mainly due to the broad range of diets used in previous studies.^7–9^ For example, the United Kingdom Prospective Diabetes Study recommended a reduced-calorie diet (50%–55% carbohydrate, 30%–35% fat, 10%–15% protein) in the intensive treatment arm, with or without intensified therapy using sulfonylurea drugs, insulin and/or metformin. Although participants reported caloric intakes lower than required and, on average, A1C was reduced by 0.9% in the intensive treatment versus conventional treatment arm, participants’ average weight also increased by 2.9 kg at the end of the study.^10^ The American Diabetes Association (ADA) currently recommends individualized meal plans that focus on nutrient-dense foods that are low in refined carbohydrates and saturated fats. However, specific amounts of carbohydrates, proteins and fats are not currently defined.^11^

In randomized controlled trials and observational studies, low carbohydrate, high fat (LCHF) diets have yielded promising improvements in glycemic control and weight loss, and often concurrently reduce the number and/or doses of antidiabetes medications.^12–24^ The LCHF diet can also improve cardiometabolic parameters such as high-density lipoprotein cholesterol (HDL), triglycerides, low-density lipoprotein (LDL) cholesterol particle size, the ratio of apolipoprotein (Apo) B to Apo A1 and the ratio of total cholesterol to HDL-C, and potentially reduced cardiovascular risk, although long-term cardiovascular outcome studies are lacking.^25–27^ The ADA recently revised its lifestyle management guidelines, stating that a very low carbohydrate diet is a feasible approach for those with hyperglycemia who wish to reduce glucose-lowering medications.^11^

Despite these favorable reports about the LCHF diet, its precise impact on metabolism is still uncertain because macronutrient proportions were inconsistent between past studies, with carbohydrates as high as 40% of total calories, potentially diluting impact.^15 17 24^ One study with a stricter definition of low carbohydrate (5%–10% of total calories), administered remotely, gave highly successful results even after 1 year.^23^ Also, to our knowledge, the LCHF diet has not been previously studied in real-world clinical settings.

In the following study, we tested the effectiveness of the LCHF diet in patients with type 2 diabetes in community-based clinical practice. We hypothesized that the LCHF diet would: (1) result in a significant reduction in mean A1C, compared with usual care (UC); (2) potentially reduce the need for antihyperglycemic agents and (3) would also improve other metabolic parameter(s) such as total body weight, fasting plasma glucose (FPG), blood pressure and lipids.

## Research design and methods

### Study design

A retrospective analysis of electronic medical records was conducted to compare glycemic and other metabolic outcomes in patients who adhered to the LCHF diet for ≥3 months, compared with patients who received usual diabetes care. The primary outcome was the change in A1C between baseline and follow-up visits. Participants had four visits: visit 1 (baseline), visit 2 (6–11 weeks), visit 3 (12–16 weeks) and visit 4 (17–21 weeks). Secondary outcomes included change in total body weight, body mass index (BMI), FPG, LDL, HDL, triglycerides and alanine aminotransferase (ALT), systolic (SBP) and diastolic (DBP) blood pressure measured at baseline and follow-up (≥12 weeks). Total body weight was measured with patients lightly clothed and without shoes. BMI was calculated by (body weight in kilograms)/(height in meters)^2^. Blood pressure was measured with a manual cuff at each visit, using standard clinical procedures. All blood tests were obtained after an 8-hour fast by either a commercial laboratory (LabCorp, Burlington, North Carolina, USA or Quest, Seacaucus, New Jersey, USA) or through Johns Hopkins Medical Laboratories (Baltimore, Maryland, USA).

### Study population and data extraction

Participants were identified from the Johns Hopkins Community Physicians (JHCP) Downtown Bethesda and Germantown Endocrinology practices from extraction of electronic medical records of patients seen between 1 January 2015 and 30 April 2018 who: 1) had a diagnosis of type 2 diabetes (defined by the 9th and 10th revisions of the International Statistical Classification of Diseases and Related Health Problems (ICD-9, ICD-10)codes for diabetes) and 2) were overweight (defined as a BMI≥25 kg/m^2^). Pregnant patients and those with stage 4–5 chronic kidney disease were excluded. The LCHF group consisted of patients who were (a) referred by the endocrinologist to a JHCP medical bariatric specialist, (b) advised on the LCHF diet and chose to follow it and (c) completed ≥3 months of follow-up. The UC group consisted of patients who did not consult the medical bariatric specialist, whether referred or not, and who received UC with at least 3 months of follow-up in endocrinology clinic.

The study team engaged with the Core for Clinical Research Data Acquisition to query clinical data for the patient cohort from Clarity database, the Structured Query Language reporting database for Epic, JHCP’s electronic medical record system. Patients were extracted using ICD-9 and ICD-10 codes for diabetes: 250.* and E11.*, respectively. Charts were queried for medical record number (for further chart review), dates of service with the endocrinologist, dates of service with the bariatric physician, date of birth, sex, ethnicity, medication list, weight, BMI, A1C, FPG, LDL, HDL, triglycerides, ALT, SBP and DBP. All patients who could be matched on age and BMI were included in the UC group to maximize sample size. A total of 49 LCHF patients and 75 UC patients were included in the final analysis. Patient selection from the extraction is detailed in online supplementary appendix 1.

10.1136/bmjdrc-2019-000980.supp1 Supplementary data

### LCHF diet

Patients in the LCHF group were instructed to bring a food log, or a list of regularly consumed foods, including drinks and snacks to their first meeting with the medical bariatric physician. Patients were educated by the bariatric physician on carbohydrate metabolism and insulin’s role in lipogenesis and weight gain, in simplified terminology. Patients were recommended to restrict net carbohydrate (total carbohydrates minus fiber) intake to ≤20 g/day or 5%–10% of their total calories, whichever was lower, as defined by Hallberg *et al*.^23^ The daily recommendation for protein was 20%–25% of total calories, based on their sex, physical activity level and ideal body weight. Recommended total fat intake was 65%–70% of total calories. Permitted food and beverages included meats, poultry, fish, eggs, low-carb nuts, seeds, non-starchy vegetables, high fat dairy products (eg, sour cream, heavy cream, cream cheese, hard cheese, plain full fat yogurt), fats and oils such as olive oil, butter, coconut oil and beverages such as water and unsweetened tea or coffee. Sample meals, snack options and recipes available online were discussed. Patients were advised to eat only when hungry and to avoid eating late at night. No caloric restriction was imposed. All patients were recommended to drink at least six to eight glasses of water per day and encouraged to keep a food log either on paper or using a free online calorie counter (eg, MyFitnessPal). Food logs were reviewed at subsequent visits to monitor diet adherence.

Before starting the LCHF diet, patients were recommended by the endocrinologist to discontinue use of sulfonylurea drugs and reduce insulin doses by 30%–50% (to avoid hypoglycemia) if they were taking these medications. Patients in both groups were offered a prescription for phentermine to aid in weight loss. All patients were encouraged to check home blood glucose readings at least once a day, preferably fasting, and keep a log. Those on multiple daily insulin injections were asked to check fingerstick blood glucose prior to meals and at bedtime. Patients on insulin were asked to return to the clinic 2 weeks after the initial visit, all other patients returned 2–4 weeks later. Subsequent follow-up visits were every 1–3 months and decided on an individual basis by the medical bariatric specialist.

Participants in the UC group were encouraged to eat high-fiber foods (such as vegetables, fruits, whole grains and legumes), low-fat dairy products, fresh fish and foods low in saturated fat. They were offered standard counseling regarding diabetes self-management and medication adjustments.

Patients in both groups were advised to increase their physical activity to at least 30 min every day, in one or multiple sessions. The importance of adequate sleep for weight management was discussed, and patients were advised to sleep 6–8 hours per night.

### Statistical analyses

Sample size calculations were based on data from Saslow *et al*.^22^ Assuming that the same number of patients are in the LCHF and UC groups, at least 48 people were needed in each group to achieve a 80% power to reject the null hypothesis of equal mean A1C, assuming the population mean A1C difference is 0.7% at follow-up with an SD for both groups of 1.2% and with a significance level (alpha) of 0.05 using a two-sided two-sample equal-variance t-test.

To compare the patient baseline characteristics between the two groups, we used the Student’s t-test or Wilcoxon rank-sum test for continuous variables, depending on the distribution on the variable; χ^2^ test or Fisher’s exact test was used for the categorical variables. The Student’s t-test was used to compare the mean A1C and the change in total body weight between the two groups at visits 1–4. The paired t-tests were used to compare the lab values at baseline and visit 3 for each group. A linear regression model with generalized estimating equations and robust SE estimates was used to calculate the mean insulin dosages at each visit and compare the mean doses at follow-up visits to the baseline visit for each group, among those patients ever taking insulin during the study. The descriptive frequencies and percentages for the change in glucose-lowering medication usage from baseline to visit 4 was calculated within each group among patients who were ever on the medications, including at baseline or initiated during the study.

To investigate differences in the A1C changes during follow-up between LCHF and UC groups, we used multivariable linear mixed effects models. Model 1 was unadjusted. Model 2 adjusted for potential confounders such as age, sex, baseline BMI, baseline A1C and baseline insulin dosage. Random intercepts were included to account for correlations due to repeated measurements from the same participant. All analyses were performed using the statistical software Stata V.15.1, with p values <0.05 considered to be statistically significant. Mean±SD was reported unless otherwise indicated.

## Results

Table 1 shows the baseline characteristics of the 49 LCHF and 75 UC patients. Although groups were matched for age, the LCHF group had a slightly younger mean age (57.3±10.2 years) than the UC group (63.1±10.9 years; p=0.004).

**Table 1:** Baseline characteristics of study participants

Characteristic	LCHF group	Usual care group	P value
n	49	75	
Sex (female)	31 (63%)	42 (56%)	0.42
Age (years)	57.3 (10.2)	63.1 (10.9)	0.004
BMI (kg/m^2^)	35.3 (7.4)	33.7 (6.0)	0.19
25–29.99	11 (22%)	21 (28%)	0.37
30–39.99	26 (53%)	43 (57%)	
>40	12 (24%)	11 (15%)	
Weight (kg)*	99.7 (27.4)	94.0 (20.4)	0.17
Systolic blood pressure (mm Hg)*	125.1 (11.6)	129.3 (17.3)	0.14
Diastolic blood pressure (mm Hg)*	75.1 (9.8)	72.8 (12.3)	0.27
A1C (%)*	8.2 (1.5)	7.9 (1.8)	0.44
Fasting plasma glucose	161.5 (56.2)	159.3 (68.3)	0.85
LDL cholesterol (mg/dL)	90.7 (28.4)	97.2 (49.0)	0.43
HDL cholesterol (mg/dL)	48.4 (13.8)	50.3 (16.8)	0.52
Triglycerides (mg/dL)	169.8 (99.4)	165.0 (98.2)	0.80
ALT (U/L)	33.3 (29.3)	26.2 (21.1)	0.14
Glucose-lowering medications			
Insulin, n (%)	21 (42.9)	36 (48)	0.57
Insulin dose (units)	64.1 (151.5)	34.7 (56.7)	0.13
Sulfonylurea, n (%)	19 (39)	24 (32)	0.44
DPP-4 inhibitor, n (%)	17 (35)	9 (12)	0.002
GLP-1 agonist, n (%)	6 (12)	4 (5)	0.19
SGLT-2 inhibitor, n (%)	5 (10)	0 (0)	0.008
Thiazolidenedione, n (%)	0 (0)	0 (0)	–
Metformin, n (%)	44 (90)	47 (63)	<0.001
Treatment category			0.01
Lifestyle only, n (%)	1 (2.0)	12 (16)	
Oral medications only, n (%)	27 (55.1)	26 (34.7)	
Oral medications+insulin, n (%)	19 (38.8)	26 (34.7)	
Insulin only, n (%)	2 (4.1)	10 (13.3)	

*Mean and SD are displayed unless otherwise noted.

ALT, alanine aminotransferase; BMI, body mass index; DPP-4, dipeptidyl peptidase-4; GLP-1, glucagon-like peptide-1; HDL, high-density lipoprotein; LCHF, low carbohydrate, high fat; LDL, low-density lipoprotein; SGLT-2, sodium-glucose transporter-2.

Mean A1C and BMI were similar between groups, and both groups had similar proportions of patients who were overweight/obese. The proportions of patients on insulin, sulfonylureas and glucagon-like peptide-1 (GLP-1) agonists at baseline were similar between groups. More patients in the LCHF group were on dipeptidyl peptidase-4 (DPP-4) inhibitors (35% vs 12% of controls, p=0.002), sodium-glucose transporter-2 (SGLT-2) inhibitors (10% vs 0% of UC group, p=0.008) and metformin (90% vs 63% of UC group, p<0.001). Relatively more patients in the UC group were on lifestyle only or insulin only, compared with the LCHF group.

**Figure 1:**
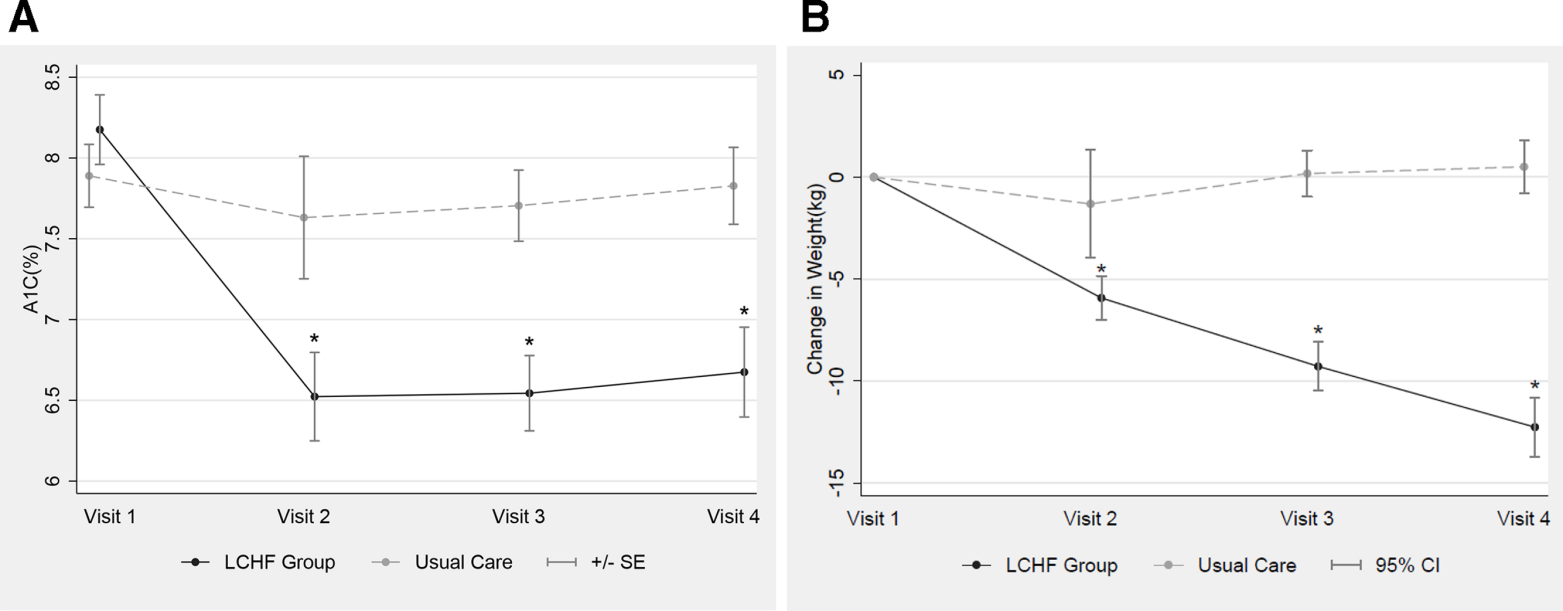
Mean change in A1C and total body weight between LCHF and UC groups during the study including: (A) change in A1C at visits 2–4 for UC and LCHF groups and (B) change in body weight (kg) at visits 2–4 for UC and LCHF groups. *P<0.001 compared with visit 1. LCHF, low carbohydrate, high fat; UC, usual care.

The LCHF group had improvement of A1C at every visit compared with the UC group, and at visit 4 had a mean A1C of 6.67% (95% CI 6.13 to 7.22) compared with a mean A1C of 7.8% in the UC group (95% CI 7.36 to 8.29); this difference was statistically significant (−1.29 (95% CI −1.75 to 0.82); p<0.001, figure 1A).

A mixed effects regression was performed to assess the ability of baseline characteristics to individually predict mean difference in A1C between the LCHF and UC groups at the end of the study (table 2). In the final, fully adjusted model, only baseline A1C was found to be a statistically significant predictor (r^2^=−0.38; 95% CI –0.49 to –0.27; p<0.001).

**Table 2:** Regression analysis modeling the relationship of dietary group (LCHF vs UC) to the change in A1C levels at each follow-up visit (visits 2–4) compared with baseline (visit 1)

	Model 1			Model 2		
	Beta-coefficient*	95% CI	P value	Beta-coefficient†	95% CI	P value
**Visit 2:** (6–11 weeks)	−1.46	−2.09 to 0.84	<0.001	−1.49	−2.07 to 0.91	<0.001
**Visit 3:** (12–16 weeks)	−1.48	−1.95 to 1.00	<0.001	−1.27	−1.70 to 0.84	<0.001
**Visit 4:** (17–21 weeks)	−1.32	−1.83 to 0.81	<0.001	−1.29	−1.75 to 0.82	<0.001

*Beta-coefficients represent the mean difference between dietary groups (LCHF vs UC) in the A1C change (A1C at that follow-up visit−A1C at baseline).

†Model 1 is unadjusted; model 2 is fully adjusted for baseline characteristics at visit 1, including sex, A1C, BMI and dose of insulin in units.

LCHF, low carbohydrate, high fat; UC, usual care.

Patients on the LCHF diet also lost significantly more weight at each visit compared with those in the UC group (figure 1B), with a mean change of −12.3 kg (p<0.001), representing a mean reduction of 11.9% of total body weight compared with baseline, at visit 4. By comparison, the UC group had a non-significant increase of 0.5 kg (p=0.4) in mean weight at follow-up (figure 1B).

Significantly more patients in the LCHF group (49%, compared with 2% in UC group) elected to use phentermine to aid in weight loss. However, in regression models that adjusted for age, sex, baseline BMI and duration of diabetes among participants in the LCHF group, no significant difference in A1C was found between patients who used phentermine versus those who did not (0.38% higher A1C in patients who took phentermine; 95% CI −0.122 to 0.879, p=0.14). Furthermore, our data show that the greatest A1C change occurred by visit 2, when only modest weight loss had occurred by this time in the LCHF group (−5.2 kg). A1C remained stable after this time point, while weight continued to decline as shown in figure 1B.

For secondary outcomes, changes in fasting glucose, lipid profile, AST and SBP and DBP between baseline and visit 3, which had the most data for both groups, were compared. If visit 3 data were unavailable, we used data from visit 4. FPG improved significantly in the LCHF group between visit 1 and visit 3 (n=27), with a mean reduction of 43.5±76.3 mg/mL (p<0.05) compared with a non-significant reduction of 8.5±8.0 mg/mL (p=0.29) in the UC group (n=62). The reduction in serum triglycerides approached significance in the LCHF group (−25.61±7.96 mg/mL, p=0.09, n=27), while there was a non-significant increase in the UC group (+18.41±159.84 mg/mL, p=0.40, n=54). Other lipid measurements remained stable in both groups, including LDL and HDL. The reduction in AST approached significance in the LCHF group (−3.70±11.02 mg/mL, p=0.09, n=27), while it remained stable in UC group (+1.67±14.47 mg/mL, p=0.397, n=55). There were no significant changes in SBP or DBP between visit 1 and visit 3 in either group.

**Figure 2:**
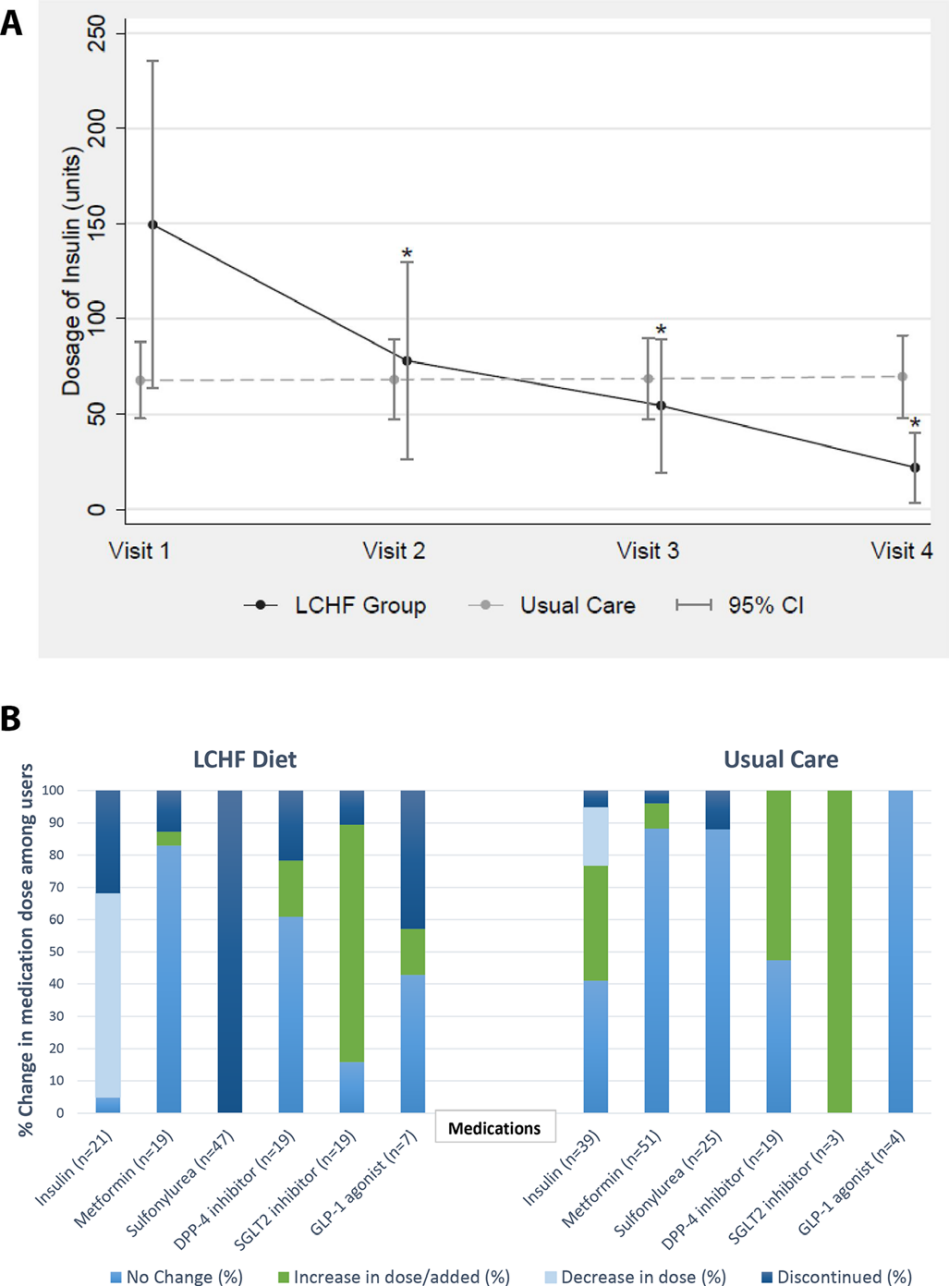
(A) Changes in mean insulin dose for patients ever on insulin during the study period. *P<0.001 when compared with visit 1. (B) Changes in in medication dosing for visit 1 to visit 4 for patients ever on medication; *n* represents the number of participants ever on the medication. DPP-4, dipeptidyl peptidase-4; GLP-1, glucagon-like peptide-1; LCHF, low carbohydrate, high fat; SGLT-2, sodium-glucose transporter-2.

The LCHF group had a reduction or discontinuation of glucose-lowering medications by their healthcare provider when clinically indicated more frequently than the UC group. The two groups had similar proportions of patients on insulin therapy at baseline (LCHF: n=21; UC: n=36) as shown in table 1. All patients in the LCHF group either had insulin discontinued (36.8%) or insulin dose reduced (63.2%) by the end of follow-up. By contrast, of the UC patients taking insulin at baseline, only 5.1% had insulin discontinued and 18.0% had insulin dose reduction by the end of follow-up; 41.0% had no change and 35.9% had an increase in insulin dose(figure 2A,B). Among those initially on this therapy, sulfonylurea medications were discontinued by the provider in all LCHF group patients to prevent hypoglycemia; by contrast, only 12% of participants in the UC group were able to discontinue this medication (figure 2B). Overall, 12.8% of those in the LCHF group initially taking metformin discontinued this medication, compared with only 3.9% of metformin-taking UC patients. Within the LCHF group, many patients also discontinued the use of DPP-4 inhibitors (21.7% of initial users), SGLT-2 inhibitors (10.5% of initial users) and GLP-1 agonists (42.9% of initial users), whereas none of the UC patients who took these medications at the baseline visit discontinued them during follow-up.

## Discussion

In our study, we analyzed a cohort of patients with type 2 diabetes who were managed in a community-based practice. Our results demonstrate that participants who followed the low carbohydrate, a high fat diet had superior glycemic reduction, as measured by A1C, compared with those who received UC at every visit. This finding was found to be significant at the end of follow-up even after accounting for age, sex, baseline BMI and insulin dosage. The improved A1C was accompanied by a significant 11.9% reduction in total body weight, with nearly 50% of patients discontinuing insulin a few months after starting the LCHF diet. By contrast, patients receiving UC had no significant change in glycemic control, non-significant changes in weight and increased insulin doses.

Our study adds to growing evidence that supports the LCHF diet in the treatment of type 2 diabetes and further demonstrates its effectiveness in real-world settings. Our results are consistent with prior studies of LCHF diets (defined as 5%–10% carbohydrates), reporting a significant reduction in A1C of >1% over a period of 12 weeks to 1 year.^14 19–21 23^ In particular, our results are most comparable to the Virta Health study,^23^ a remotely monitored intervention that implements the LCHF diet in patients with type 2 diabetes. At 1 year, the LCHF group showed a significant difference in A1C (−1.5%±0.2% (p<0.05)), comparable to similar results in our study over ≥3 months. LCHF patients in the Virta Health study also reduced or discontinued insulin and most other glucose-lowering medications, and consequently reduced the mean annual cost of medications per person by 46% over the first year on the LCHF diet. We found a similar reduction in glucose-lowering medications, supporting the hypothesis that the LCHF diet has the potential to improving patient outcomes and reduce costs. Americans spend about US$106 billion per year on diabetes prescription medications and supplies alone; this and other factors including the rising cost of insulin and its accessibility can directly impact daily care for patients with diabetes.^28^ Patients in our study had clinic visits covered by insurance. By contrast, virtual or remote LCHF programme cost each patient thousands of dollars, if not covered by their insurance^29^ and may be financially unfeasible for many patients. Thus, our study demonstrates the feasibility of implementing the LCHF diet in a community-based practice as part of an ongoing dietary treatment plan for the management of type 2 diabetes.

Baseline A1C, insulin use and duration of diabetes are often used as surrogate measures of diabetes severity, and used as predictors for partial or complete remission of diabetes after bariatric surgery.^30^ Similarly, our study demonstrates that higher A1C at baseline predicts a greater improvement in A1C during follow-up with the LCHF diet, while insulin dose was not related. Information regarding the duration of diabetes was not available for all patients in our study, but would be an important predictor to investigate in future studies.

Mechanisms for the improved glycemia observed with the LCHF diet include dietary carbohydrate restriction, lessening the need for endogenous insulin secretion and exogenous insulin administration as well as resulting in subsequent weight loss.^31–33^ By following an LCHF diet and restricting carbohydrate intake, plasma glucose levels decrease and accordingly, overall insulin levels are reduced, allowing for lipolysis and the use of non-esterified fatty acids as an alternate fuel source. Overall, a state of mild, physiological ketosis is induced.^34^ The reduction in hyperglycemia occurs often within days of starting on the LCHF diet, much before significant weight loss is observed.^34^

Despite studies demonstrating the efficacy of the LCHF diet in managing type 2 diabetes, its long-term benefit remains unclear. As such, the 2019 American Diabetes Association Guidelines on lifestyle management of patients with type 2 diabetes, while acknowledging the modest benefit in A1C reduction with LCHF diets, do not necessarily recommend for or against its implementation.^11^ The main uncertainty for any dietary intervention is that patients will, over time, revert to their previous lifestyle habits. For the LCHF diet in particular, other uncertainties include differing definitions of the LCHF diet, and how it is implemented. A recent meta-analysis of 36 studies by van Zuuren *et al*,^35^ including 33 randomized controlled trials and 3 case-control trials, compared the LCHF diet with a traditional low fat diet. This study found that the LCHF diet caused a significant reduction in A1C of −1.38% (95% CI −2.64% to −0.11%) in the first 8 weeks, but the mean difference in A1C was attenuated by 8–16 weeks (−0.55% (95% CI −0.93 to 0.17)) and onwards, through 26 weeks. Among these studies, the majority allowed ~40% carbohydrates, whereas only two defined ‘low carbohydrate’ as 5%–10% carbohydrates (or <20 g of carbohydrates) per day. Thus, the potential long-term effectiveness of the 5%–10% carbohydrate LCHF diet remains an area for future research.

Although studies of the long-term cardiovascular outcomes of the LCHF diet are lacking, there is evidence that key biomarkers of cardiovascular disease are improved, including serum triglycerides and HDL-C, which often correlate with improved hyperglycemia. We saw a non-significant reduction in triglycerides. Values for HDL-C either increased or remained stable in previous heterogeneous studies of the LCHF diet.^15 17 18 20 22 23^ We found no significant changes in HDL in either group, but note that both groups had initial HDL values in the normal range. We also found no significant changes in LDL levels in either group, consistent with previous studies.^15 17 18 20 22 23^ Two previous studies associated LCHF diets with the more favorable distribution of LDL particles (more non-atherogenic, large LDL particles^25 27^), although we did not measure this parameter. While controversial, some studies suggest patients who take insulin may have a dose-dependent increased risk of cardiovascular events.^36–39^ The possible increased cardiovascular morbidity may be related to weight gain and hypoglycemic events, which can accompany the use of sulfonylurea drugs and insulin particularly at higher doses. Highlighting the potential beneficial impact of the LCHF diet, in which sulfonylureas were routinely discontinued and insulin doses were initially reduced, and many of our patients in the LCHF group were able to further reduce or eliminate insulin.

To our knowledge, this is the first study to assess the LCHF diet in a community-based, ‘real-world’ setting while also collecting data on multiple metabolic parameters and over multiple visits, making the results generalizable. An important strength of our study was our adherence to a rigorous definition of LCHF macronutrient distribution of <20 g of carbohydrates (or <5%–10% of total calories) daily. In addition, patients kept detailed food logs that were regularly reviewed by a multidisciplinary team of healthcare providers to confirm adherence to the LCHF diet at each visit.

Our study also has important limitations. First, other factors may have contributed to the observed differences in A1C reduction between the LCHF and control groups. Notably, since this was not a randomized study, the LCHF patients were self-selected and may have been more motivated to comply with lifestyle intervention. However, this further underscores the potential benefits of healthcare providers discussing the LCHF diet as an option to their patients in clinical practice. The LCHF patients also had more face-to-face time with a healthcare provider than the UC group, due to the recommended bimonthly or monthly visits with the bariatric physician, which may have impacted their outcomes. Lastly, about half of the LCHF group elected to start phentermine which may have impacted weight; however, we found that A1C change was similar in participants who used phentermine compared with those that did not among LCHF patients. Future long-term studies to gain further metabolic insights into the LCHF diet are needed, including verification of nutritional ketosis with either serum or urinary ketone measurements while following the diet.

In summary, our study demonstrates that it is feasible and safe to implement the LCHF diet in a ‘real-world’ community practice setting among patients with type 2 diabetes, and that this diet may offer superior glycemic reduction, along with greater weight loss, compared with UC. The potential to reduce glucose-lowering medications including insulin may ultimately also help lower the personal and societal costs associated with type 2 diabetes. Although further studies are needed to evaluate the LCHF diet’s long-term efficacy and cardiovascular benefits, our results add to growing evidence that the LCHF diet in motivated patients may be a practical and effective method to improve glycemic control with several additional metabolic benefits, and should be considered as a viable treatment option in the management of type 2 diabetes.
