# Supplementary material for: Effects of the low carbohydrate, high fat diet on glycemic control and body weight in patients with type 2 diabetes: experience from a community-based cohort
Source: BMJ Open Diabetes Res Care. 2020 Mar 18;8(1):e000980. doi: 10.1136/bmjdrc-2019-000980 (PMC7103851; doi:10.1136/bmjdrc-2019-000980)
Supplement: Supplementary data [file bmjdrc-2019-000980supp001.pdf]

Appendix 1. Study flowchart describing identification and selection process of patients for LCHF and UC groups.

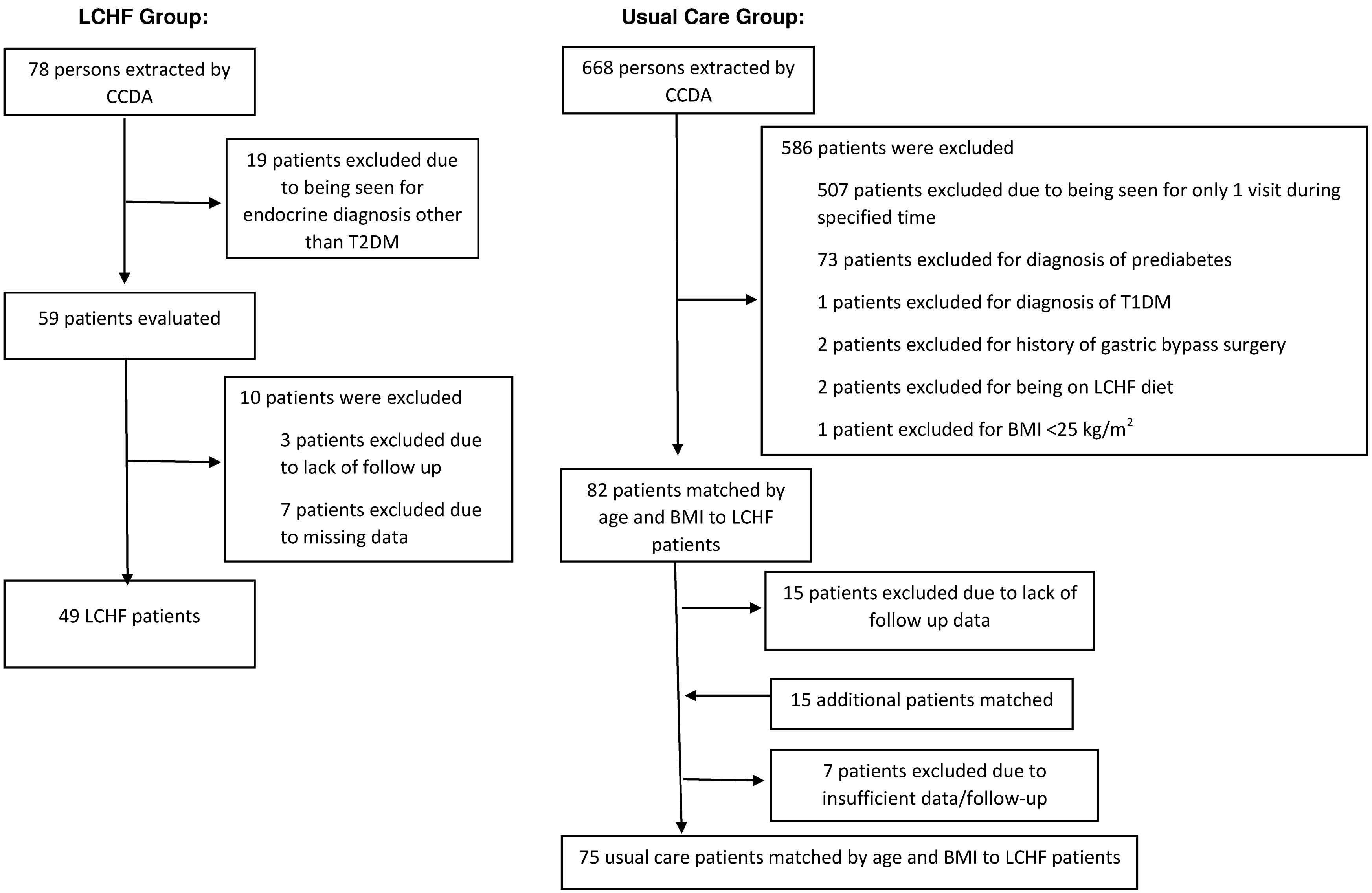

Appendix Figure 1. Study flowchart describing identification and selection process of patients for LCHF and UC groups. Abbreviations: T2DM: type 2 diabetes mellitus; T1DM: type 1 diabetes mellitus; BMI: body mass index; LCHF: low carbohydrate, high fat , UC: Usual Care
